# Effects of Environmental Gas and Trace Water on the Friction of DLC Sliding with Metals †

**DOI:** 10.3390/mi8070217

**Published:** 2017-07-11

**Authors:** Yoshihiro Kurahashi, Hiroyoshi Tanaka, Masaya Terayama, Joichi Sugimura

**Affiliations:** 1 Department of Hydrogen Energy Systems, Graduate School of Engineering, Kyushu University, Fukuoka 819-0395, Japan; te111729@gmail.com; 2 Department of Mechanical Engineering, Kyushu University, Fukuoka 819-0395, Japan; tanaka.hiroyoshi.315@m.kyushu-u.ac.jp (H.T.); terayama.masaya.629@m.kyushu-u.ac.jp (M.T.); 3 Research Center for Hydrogen Industrial Use and Storage, Kyushu University, Fukuoka 819-0395, Japan; 4 International Institute for Carbon-Neutral Energy Research, Kyushu University, Fukuoka 819-0395, Japan

**Keywords:** DLC, friction, gas, trace water, aluminum, FT-IR, laser Raman spectroscopy

## Abstract

This paper describes an experimental study on the friction of a-C:H diamond-like carbon (DLC) and ta-C DLC coatings in gas with different concentration of trace water. Pin-on-disk sliding experiments were conducted with DLC coated disks and aluminum pins in hydrogen, nitrogen, and argon. Trace oxygen was eliminated to less than 0.1 ppm, while water in the gas was controlled between 0 and 160 ppm. Fourier transform infrared spectroscopy (FT-IR) and laser Raman spectroscopy were used to analyze the transfer films on the metal surfaces. It was found that trace water slightly increased friction in hydrogen gas, whereas trace water caused a significant decrease in the friction coefficient in nitrogen and argon, particularly with a-C:H DLC. The low friction in hydrogen was brought about by the formation of transfer films with structured amorphous carbon, but no differences in the structure and contents of the films were found in the tests with and without trace water. In nitrogen and argon, the low friction with a-C:H DLC was achieved by the gradual formation of transfer films containing structured amorphous carbon, and FT-IR spectra showed that the films contained CH, OH, C–O–C, and C–OH bonds.

## 1. Introduction

Diamond-like carbon (DLC) is one of the most successful materials for tribological coatings. Initially, DLCs were introduced for their low friction and high wear resistance, although only in particular environments and under limited sliding conditions. After decades of studies and developments, DLCs now come in a wide variety of types with different manufacturing processes and compositions, and can be used in severe practical conditions from dry contacts to oil lubricated contacts [1,2,3]. Examples include valve trains, piston and piston rings in automotive engines, magnetic storages, cutting tools, and components in other engineering and medical fields [4,5]. DLCs are important candidates in the context of reducing energy consumption and CO_2_ emissions through low friction and wear loss.

The low and super-low friction exhibited by DLC strongly depends on their atomic structure, the formation of carbon transfer layers, the presence of hydrogen and the nature of the environments in which sliding takes place. Many of the earlier studies on undoped DLC revealed that hydrogen content in DLC gave rise to diverse frictional behaviors in different gas environments.

Hydrogen containing DLC generally showed low friction coefficient in vacuum and in inert gas, such as nitrogen and argon, while it showed high friction in the presence of water and oxygen. The decrease in friction was associated with the formation of carbon transfer films with a disordered graphitic structure on the counterface materials [6,7,8,9,10]. Formation of carbon transfer films is crucial for obtaining a low friction coefficient of less than 0.1. With hydrogenated DLC, a low friction coefficient was provided by the formation of carbon transfer layers on silicon nitride in dry argon [6], on AISI 440C steel in air after prolonged sliding [7], on AISI 52100 steel at high vacuum [8], on M50 steel dry argon [9], and on magnesia/partially-stabilized zirconia [10].

In contrast, hydrogen-free DLC exhibited very high friction in inert environments, but showed a decrease in friction by the introduction of some chemically-active species into the environment [11,12,13,14,15,16,17]. The coefficient of friction was as high as 0.6 in vacuum, nitrogen, and argon, which was attributed to strong covalent bond interactions between carbons on the DLC surfaces. This interaction was attenuated somehow by the introduction of hydrogen and oxygen, and most significantly by water vapor; the coefficient decreased to less than 0.1 by 100 Pa of water vapor [13]. Another study on hydrogen-free DLC against aluminum alloy and tungsten carbide showed that the friction coefficient was high in vacuum, nitrogen, and dry air, while it was around 0.1 in air with relative humidity higher than 20% with the formation of carbonaceous transfer layer [15].

It was believed that, for either type of DLC, passivation of the carbon surface by adsorption of some species, including hydrogen and water, could eliminate the covalent bond interactions between carbons to yield lower friction. Donnet et al. showed that the friction coefficient for DLC with lower hydrogen content against AISI 52100 steel increased to as high as 0.6 in ultrahigh vacuum and in argon, while it decreased to less than 0.01 when hydrogen gas of pressure of 10 hPa was introduced in the test chamber [18]. As this behavior was similar to the super-low friction of DLC with higher hydrogen content, they concluded that hydrogen saturation on the carbon surfaces suppressed the carbon-carbon interaction which otherwise gave rise to higher friction. Erdemir showed that DLCs with higher hydrogen content exhibited lower coefficient of friction in dry nitrogen, and suggested that the super-low friction with highly hydrogenated DLC was provided by the elimination of the strong carbon-carbon interactions by hydrogen termination, in addition to the stronger shielding by di-hydration and repulsive electrostatic forces between hydrogen-terminated surfaces [11,14]. Fontaine et al. suggested that, while hydrogen loss from the DLC surface occurred under sliding, hydrogen termination could be restored through introduction of hydrogen in surrounding gas [19]. Gao et al. also reported that removal and replenishment of hydrogen resulted in the increase and decrease in friction, respectively, and that water had reverse effects [20]. All these works suggested that the hydrogen termination of the top surface layer of carbon atoms was the primary mechanism for super-low friction, and the supply of hydrogen to the surface either from inside the DLCs or from the environment was crucial for maintaining the low friction.

Surface analysis using laser Raman spectroscopy, X-ray photoelectron spectroscopy (XPS), Fourier transform infrared spectroscopy (FT-IR), and secondary ion mass spectroscopy (SIMS) gave supportive evidence to the hypothesis of the carbon transfer films and hydrogen termination [21,22,23,24,25,26]. These studies indicated that different structures were formed in different environments. Disordered graphitic carbon was detected by Raman spectroscopy, while the presence of hydrogen was most clearly shown by TOF-SIMS [21,25,26].

In addition to the role of hydrogen, researchers paid attention to the effect of humidity as described above [6,11,12,13,15,16,20,22,23,24]. Further studies showed again that hydrogenated DLC showed higher friction under higher humidity, and this was ascribed to the oxidation of carbon films [27,28,29,30]. On the other hand, hydrogen free DLC showed a rather low coefficient of friction in humid argon due to the formation of a carbon transfer layer [31].

Advances in molecular simulations have made it possible to examine the proposed mechanisms of hydrogen termination and super-low friction of DLC. Calculations based on the density function theory showed repulsive interactions due to hydrogen termination [32], dissociation and chemisorption of hydrogen on a diamond surface [33], and lower friction of a diamond against iron surface by H or OH termination [34]. Additionally, molecular dynamics simulations showed carbon transfer film formation and the hydrogen effect [35], and faster passivation of surface carbon and run-in by DLC with higher hydrogen content [36]. More recently, Hayashi et al. investigated the effect of hydrogen atoms on carbon transfer films with molecular dynamics and tight-binding quantum chemistry calculations, and showed surface hydrogen atoms suppressed the formation of C–C bonds, helped breaking C–C bonds and increased the distance between DLC surfaces to provide lower friction [37]. Bai et al. conducted molecular dynamics simulations and showed that hydrogen atoms were concentrated near the surfaces to cause stronger passivation at low temperature, and they diffused into DLC to enhance graphitization at high temperature [38]. Recent studies showed larger repulsive forces and lower friction by fluorine termination than hydrogen termination [39,40]. The molecular simulations have, thus, made progress to provide useful information about the basic mechanisms, the actual complicated processes, such as those with humidity and counterface materials, still remain unsolved.

In recent years, hydrogen is gaining more and more attention as an ultimate clean energy carrier, and industries accelerate the development of systems for generation, storage, transportation, and utilization of hydrogen. DLC is a strong candidate material for tribological components in these systems because of its favorable friction and wear performance with hydrogen. With this background, some studies focused on the behavior of DLC in hydrogen gas [41,42,43,44,45].

In hydrogen systems for fuel cells, hydrogen gas must be pure enough in order to avoid the degradation of the electrode catalyst. According to an ISO standard for hydrogen for fuel cell vehicles, the concentration of contaminants, such as oxygen and water, in hydrogen should be less than several ppm [46]. This means that design of tribological elements has to take into account the purity of hydrogen. In addition to hydrogen, there are many cases where a certain degree of purity of operating gas is required, and friction and wear of materials are sensitive to a small amount of impurities in the gas.

As described above, friction and wear of both hydrogenated and hydrogen-free DLC strongly depend on water content in gas. However, in most of the studies, the water concentration in ‘wet’ gas was high in the relative humidity range from several percent up to 100%, while the water concentration in ‘dry’ gas was not clearly defined. Therefore, it is necessary to investigate tribological behavior of DLC in environments where the amount of water in gas is accurately controlled at ppm level.

The objective of the present study is to investigate how a small amount of water of ppm level in the environmental gas affects friction and wear of two types of DLC. Hydrogenated DLC and hydrogen-free DLC are tested in hydrogen, nitrogen, and argon with well-controlled water concentration.

## 2. Materials and Methods

Pin-on-disk experiments were conducted in purity controlled gas environments with DLC-coated disks and aluminum pins. The DLC coatings used in this study were a-C:H hydrogenated DLC and ta-C hydrogen-free DLC. They were coated by Nippon ITF, Inc. (Kyoto, Japan) on AISI 52100 bearing steel disks of 25 mm diameter and 4 mm thickness. The a-C:H DLC was made by the plasma enhanced chemical vapor deposition, and the ta-C DLC was made by the arc ion plating. The properties of the DLC coatings are shown in Table 1.

The pin specimens were made of pure aluminum. Their diameter was 4 mm, and they had rounded tips with a radius of curvature of 4 mm. Prior to the tests, the pin specimens were polished with emery paper and then mirror finished by using 3 μm diamond slurry. Surface roughness of the pin specimens was 0.01 μm Ra. The pin and disk specimens were cleaned in an ultrasonic bath with an acetone and hexane mixture.

**Figure 1:**
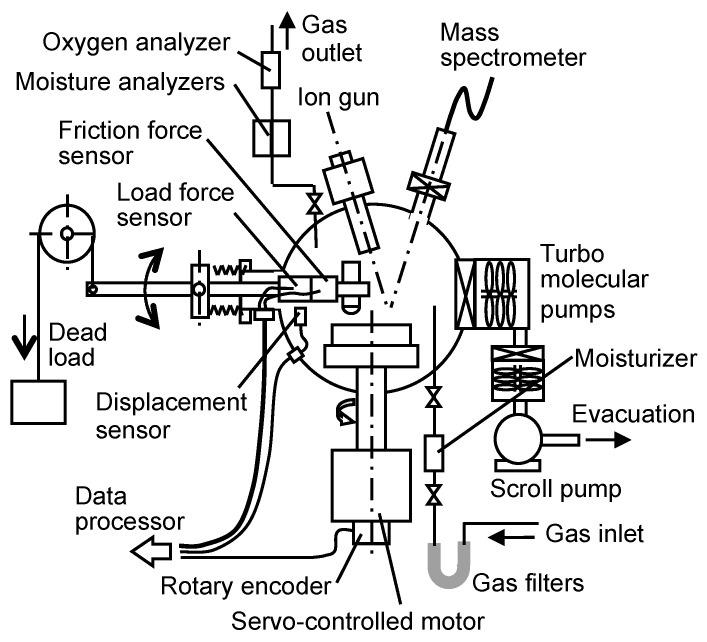
Pin-on-disk apparatus with an advanced gas control system.

Figure 1 schematically shows a pin-on-disk apparatus [47]. This has an ultra-high vacuum chamber with heaters and a temperature control system, two turbo-molecular pumps, gas filters, a moisturizer, two moisture analyzers and an oxygen analyzer. In the chamber, the disk specimen is mounted on a rotating shaft and the pin specimen is pressed against the disk specimen by a loading lever. Temperature in the chamber can be controlled in a range between 293 K and 473 K. The gas supplied is hydrogen or inert gas of 99.999% purity, and the gas filters further remove water, oxygen, and carbon monoxide such that the concentrations of oxygen and water in the filtered gas are less than 1 ppb. The moisturizer designed by Fukuda employs a permeation method that allows a small amount of water vapor to be added to gas; it has an elastomer pipe in a cylinder filled with distilled water, and water slowly penetrates through the elastomer to diffuse into gas that flows in the pipe [47]. A Panametrics Moisture Monitor, Series 35, (GE, South Burlington, VE, USA) is used to monitor water concentration greater than 1 ppm. Another moisture analyzer HALO, (Tiger Optics, LLC, Warrington, PE, USA), is a cavity ring-down spectroscopy-type analyzer to determine water concentration smaller than 1 ppm. The oxygen analyzer is a coulometric oxygen meter DF-550E, Delta F Corporation (Woburn, MA, USA). It can determine the oxygen concentrations down to 1 ppb. With these filters, the moisturizer and the analyzers, the concentration of water vapor and oxygen can be controlled in a wide range from ppb to several tens of ppm [47].

The gases used were hydrogen, nitrogen, and argon. The water and oxygen contents in the chamber were controlled in the following way: After the specimens were set in the chamber, the chamber was evacuated to a pressure level of 1 × 10^−5^ Pa. For the tests with trace water and oxygen less than 1 ppm, the chamber with the specimens was baked at 353 K for 50 min and then at 403 K for 50 min. The pressure in the chamber reduced to 1 to 3 × 10^−6^ Pa by this baking process. After the chamber was cooled down to room temperature, the test gas purified with the filters was introduced in the chamber until the pressure in the chamber reached the atmospheric pressure. The purified gas was then supplied and allowed to be exhausted at a flow rate of 2 L/min, and after confirming that the concentrations of both water and oxygen were less than 100 ppb, sliding tests were conducted. During the sliding tests, the concentrations of water and oxygen were continuously monitored.

For the tests in gas with trace oxygen less than 1 ppm and trace water larger than 1 ppm, the gas filtered and then moisturized at a predetermined water concentration was introduced into the chamber after the pressure in the chamber reached 1 × 10^−5^ Pa by evacuation. The gas was supplied and exhausted at a flow rate between 0.1 L/min and 0.5 L/min, and after confirming that the water concentration kept the predetermined level, sliding tests were conducted. With this method, the water concentration can be set between 1 ppm and 160 ppm, while keeping the oxygen concentration below 1 ppm.

All the sliding tests were conducted at 298 K in dry contact conditions. The sliding speed was 0.0628 m/s and the load was 10 N. The sliding distance was 126 m. Absolute pressure of the test gas during the tests was 0.12 MPa. The concentration of trace water was set between zero and 160 ppm, while that of trace oxygen was kept below 0.2 ppm. During the sliding tests the friction force was measured. After the sliding tests, the surfaces of the specimens were observed and analyzed with optical microscopy (Nikon, Tokyo, Japan), Fourier transform infrared spectroscopy (Thermo Fisher Scientific, Waltham, MA, USA) and laser Raman spectroscopy (Thermo Fisher Scientific).

## 3. Results

### 3.1. a-C:H Hydrogenated DLC

**Figure 2:**
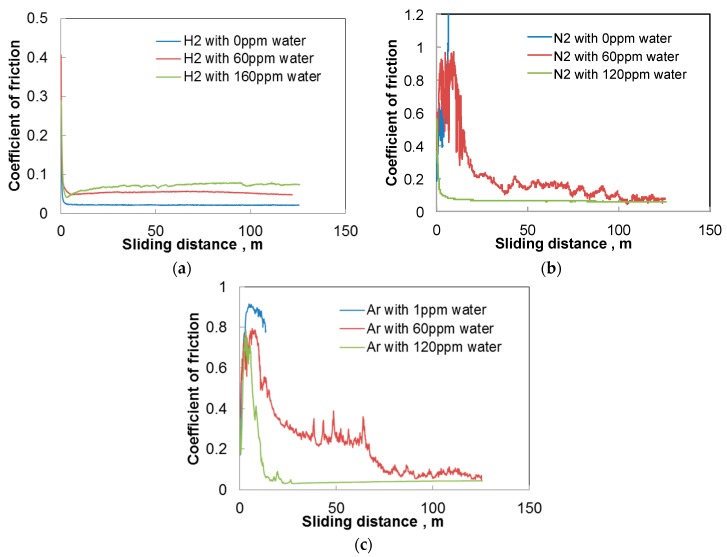
Changes in the coefficient of friction with sliding distance; a-C:H DLC disk and aluminum pin; (**a**) in hydrogen; (**b**) in nitrogen; and (**c**) in argon.

Figure 2a shows the variation of the coefficient of friction of a-C:H DLC tested against aluminum in hydrogen. The concentration of trace water in the hydrogen is shown in the figure. “0 ppm” indicates that the concentration is less than 0.1 ppm. The coefficient of friction is high at the start of sliding, but rapidly decreases below 0.1 and stabilizes. The steady state coefficient of friction is lower for smaller water concentrations. However, a small amount of water has the opposite effect in nitrogen and argon as shown in Figure 2b,c. Friction is low and stable for 120 ppm water, but it increases rapidly at the start of sliding in nitrogen with 0 ppm water and in argon with 1 ppm water. At an intermediate concentration of 60 ppm, the coefficient of friction decreases with time, but much more slowly.

These results suggest that trace water increases friction in hydrogen, but acts to reduce friction in nitrogen and argon. The increase of friction by water is similar to those found in near vacuum with low partial pressure of water by Andersson et al. [13] and Kim et al. [28]. On the other hand, the decrease of friction by water vapor in nitrogen and argon is different compared to other studies on humidity effects, where higher humidity causes an increase in the coefficient of friction [6,23,27,29,30]. However, these studies employed larger amounts of water compared to the present trace water of ppm order.

**Figure 3:**
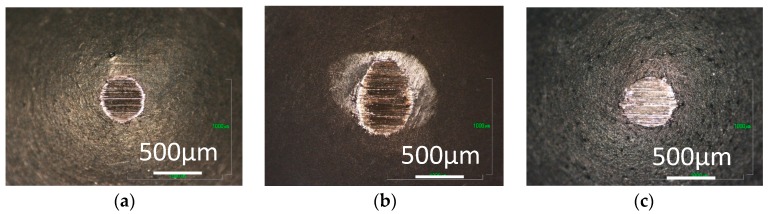
Photographs of aluminum pins slid against a-C:H DLC in hydrogen; with (**a**) 0 ppm water; (**b**) 60 ppm water; and (**c**) 160 ppm water.

**Figure 4:**
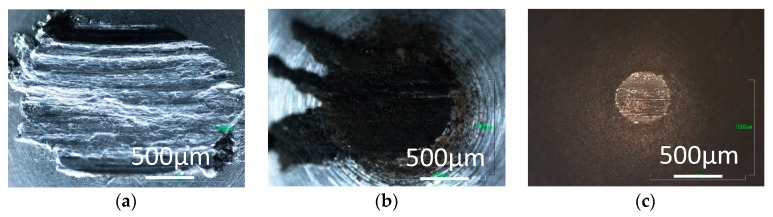
Photographs of aluminum pins slid against a-C:H DLC in nitrogen; with (**a**) 0 ppm water; (**b**) 60 ppm water; and (**c**) 120 ppm water.

**Figure 5:**
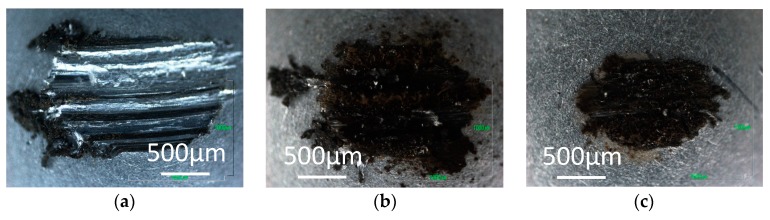
Photographs of aluminum pins slid against a-C:H DLC in argon; with (**a**) 1 ppm water; (**b**) 60 ppm water; and (**c**) 120 ppm water.

Figure 3, Figure 4 and Figure 5 show optical images of the aluminum pins after the tests. A black transfer film can be seen on the aluminum pin tested in hydrogen with 0 ppm water, as shown in Figure 3a. This is structured amorphous carbon. Carbon transfer film is also found on the pin tested with 60 ppm and 160 ppm water. It appears wear is slightly larger with trace water.

In contrast, the transfer film is not found on the pins tested in 0 ppm water in nitrogen and argon, but the pins are severely worn as shown in Figure 4a and Figure 5a. With 60 ppm and 120 ppm water, carbon transfer films and wear debris of carbon are observed on the pins tested in nitrogen and argon. More wear debris are found for the tests in 60 ppm water than those in 120 ppm, suggesting that DLC surface wears significantly during the high friction period before the coefficient of friction settles down. The wear behaviors indicate that, during that high friction period, the trace water in the inert gases has somehow contributed to the formation of carbon transfer film.

**Figure 6:**
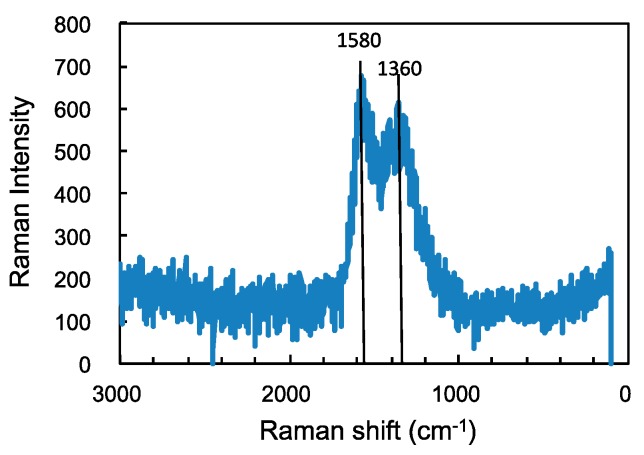
Raman spectrum taken on aluminum surface slid against a-C:H DLC in nitrogen with 60 ppm water.

Figure 6 shows one of the Raman spectrum measured on the pin tested in nitrogen with 60 ppm water, displaying the G-band and D-band peaks of the structured amorphous carbon. The similar Raman spectrum with the peaks are found in the tests in hydrogen, and in the test in the inert gases with finite amount of trace water where the coefficient of friction decreases to less than 0.1. This indicates the formation of carbon transfer films has contributed to the reduction in friction. However, these band intensities and their ratio, *R*-value, are different from location to location on the same wear scar, suggesting that the structure of the carbon films is not uniform. It is not clear from the present measurement how they distribute on each of the wear scar, and it is difficult to find a clear relationship between the Raman spectrum and the values of the coefficient of friction.

**Figure 7:**
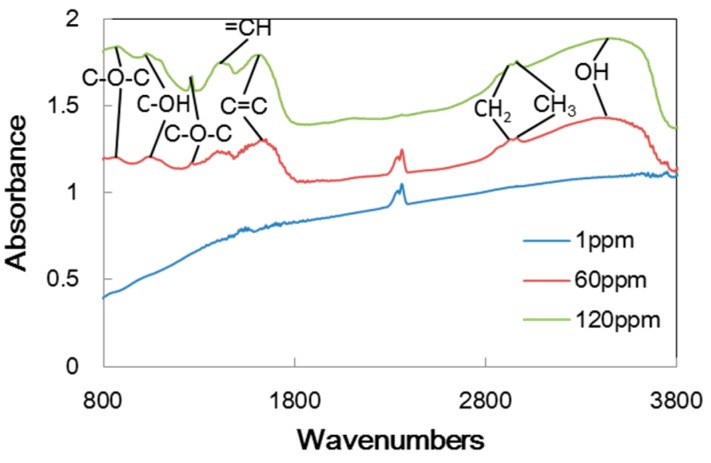
FT-IR spectrum of aluminum surface slid against a-C:H DLC in argon.

In order to look at the contents of the carbon transfer films, FT-IR analysis was made. Figure 7 shows the FT-IR spectrum measured on the transfer films generated on the pin specimen tested in argon with three different concentrations of trace water. The peaks found at 2840 and 2920 cm^−1^ show C–H bonds corresponding to straight-chain hydrocarbons. The peak at 3400 cm^−1^ indicates the presence of OH bonds, while the peaks below 1300 cm^−1^ are related to C–O–C and C–OH bonds. These peaks appear in the tests with 60 ppm and 120 ppm water, but not in the test with 0 ppm water. This implies that trace water generates H and OH species, which participate in the formation of the carbon transfer film on the aluminum pin. Once a stable film is formed, it provides constant low friction due to the hydrogen terminations at the surface.

Table 2 summarizes the peaks found in FT-IR spectra on the aluminum pin for all the tests. The symbol “○” indicates that the presence of a peak while “×” is for no peak. In the tests in hydrogen, there is only a C–H bond irrespective of the water concentration. In the tests in nitrogen and argon with no water, no peaks are found because there is no carbon film. In nitrogen with trace water of 60 ppm and 120 ppm, OH bonds may indicate water has been involved in the formation of the transfer film. In argon with trace water of 60 ppm and 120 ppm, C–O–C and C–OH bonds suggests that water has participated more actively in chemical processes involved in the transfer film formation. It is likely that the carbon transfer film is not formed in the inert gases without these processes.

### 3.2. ta-C hydrogen Free DLC

**Figure 8:**
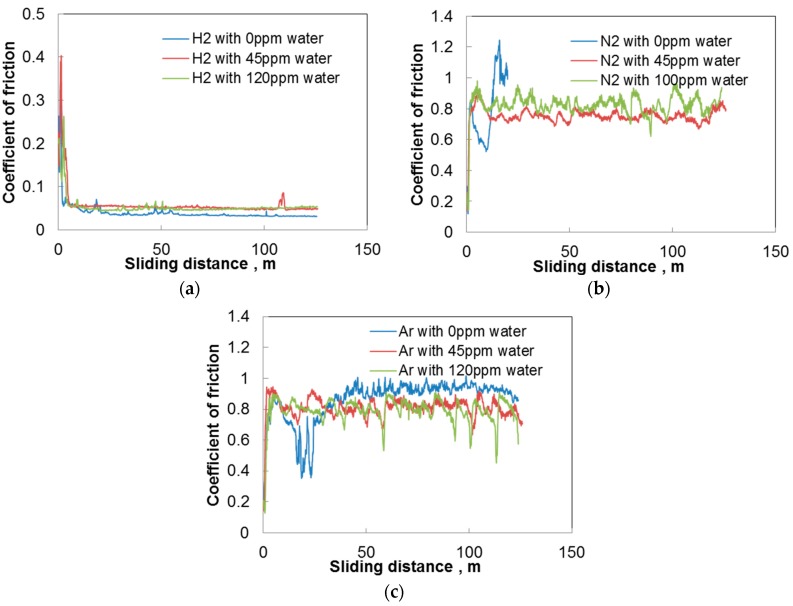
Changes in the coefficient of friction with sliding distance; ta-C DLC disk and aluminum pin; (**a**) in hydrogen; (**b**) in nitrogen; and (**c**) in argon.

Figure 8 shows the coefficient of friction of ta-C DLC tested against aluminum in hydrogen, nitrogen, and argon. The coefficient of friction is as low as 0.05 and stable in hydrogen and slightly increased by 45 ppm and 120 ppm water. The increase is smaller than in the case with a-C:H DLC. Friction is very high over 0.8 in nitrogen and argon irrespective of water concentration, though the coefficient is slightly lower with trace water than without water.

**Figure 9:**
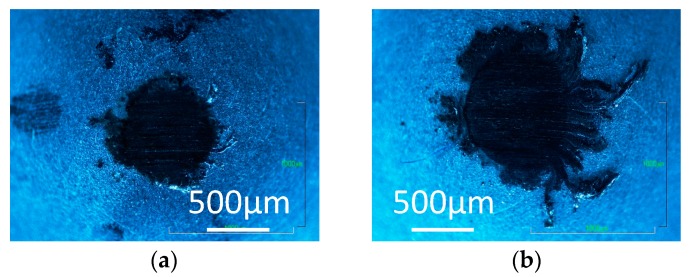
Photographs of aluminum pins slid against ta-C DLC in hydrogen; with (**a**) 0 ppm water; and (**b**) 120 ppm water.

**Figure 10:**
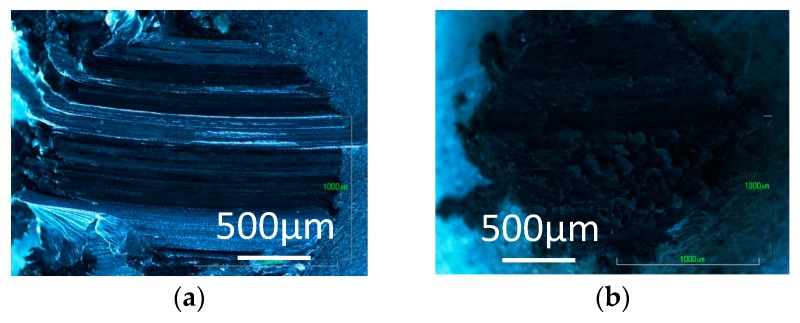
Photographs of aluminum pins slid against ta-C DLC in argon; with (**a**) 0 ppm water; and (**b**) 120 ppm water.

Optical images of the aluminum pin tested against ta-C DLC are shown in Figure 9 and Figure 10 for tests in hydrogen and argon. The carbon transfer film is observed on the pin tested in hydrogen, but wear appears more severe in 120 ppm water, than in the case of a-C:H DLC. In argon with 0 ppm water, the aluminum pin wears severely and no carbon film is formed, while the pin is covered with wear debris and carbon transfer film with 120 ppm water. The similar behaviors are observed in the tests in nitrogen. The results suggest that water assists the formation of a carbon transfer film in the inert gases as in the case of a-C:H DLC, but the film serves to only slightly reduce the coefficient of friction. The observation of the DLC disk surface has revealed that there is a transfer of aluminum to the DLC surface in the tests in nitrogen and argon, which must have occurred from the beginning of sliding and contribute to the very high coefficient of friction.

**Figure 11:**
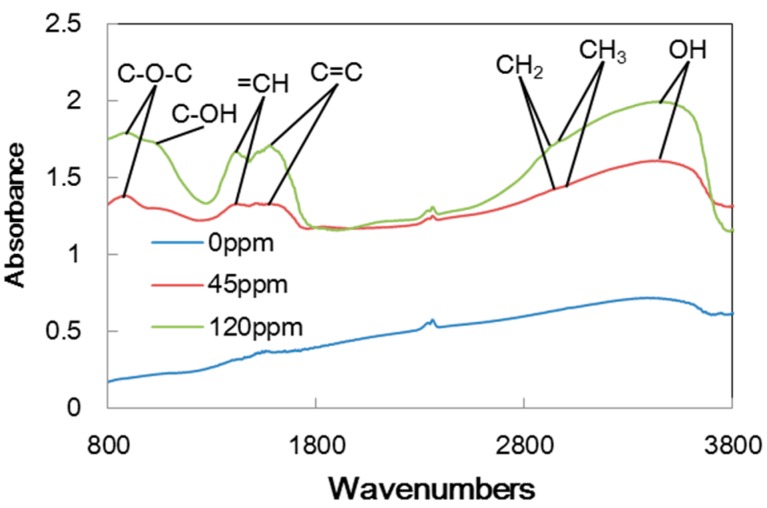
FT-IR spectrum of the aluminum surface slid against ta-C DLC in argon.

Figure 11 shows the FT-IR spectra of the transfer films produced on the pin specimens tested in argon at the three different concentrations of trace water. Similar to the case with a-C:H DLC, almost no peaks, except for hydrocarbon, are found on the pin for 0 ppm water, while hydrogen- and oxygen-related substances are present in the transfer film generated in argon with 45 ppm and 120 ppm water. Table 3 shows the summary of peaks in FT-IR spectra. In general, the contents of carbon films in inert gases with trace water are similar to those found in the tests with a-C:H DLC except that C–O bonds are also found in nitrogen, although the frictional behaviors are different from those with a-C:H DLC.

## 4. Discussion

The present experiments demonstrate that trace water in environmental gas plays an important role in the formation of carbon transfer films on the aluminum surface, which leads to different frictional behaviors depending on the gas species.

In hydrogen gas, the a-C:H DLC forms efficient carbon transfer films on the aluminum surface in a short sliding time and provided stable, low friction when water was not present. This suggested that hydrogen played a major role in the formation of a low friction carbon-carbon tribo-interface as described in the literature [11,14,18,19]. Trace water interfered with this process, either in the formation of the carbon transfer film or through the termination of surface carbon bonds with non-hydrogen groups. Therefore, the coefficient of friction gradually increased with the increasing concentration of trace water. In this study, however, no clear difference in the graphitic structure of carbon films could be found for different water concentration in Raman spectra. Additionally, no difference was identified in the FT-IR spectra; they showed only CH bonds both for the tests with and without trace water.

On the other hand, the effect of trace water on the increase of friction with ta-C DLC in hydrogen was not as significant as a-C:H DLC. This may imply that a small amount of water had less influence on the transfer film formation. The FT-IR spectra also indicated no oxygen in the carbon transfer films. The collaborative and competitive actions of hydrogen and water must be investigated further.

In nitrogen and argon gas, the influence of trace water on the coefficient of friction for both DLCs was entirely different. The a-C:H DLC exhibited a very high coefficient of friction without water, while the coefficient decreased in the presence of trace water. At a water concentration of 120 ppm, the coefficient of friction decreases to about 0.05 in argon. At 60 ppm water, the coefficient of friction was gradually reduced but showed significant fluctuation. This was brought about by wear and the formation of carbon transfer films during the relatively long sliding time. It is likely that H and/or OH help the detachment of carbon from the DLC and chemical reactions for deposition on the counter surface to enhance the transfer film formation.

The films contained a variety of groups that included carbon, hydrogen and oxygen as shown in Figure 7 and Table 2. It should be noted that the transfer films in nitrogen and in argon are slightly different; those in nitrogen contained CH and OH bonds in nitrogen, while those in argon contained C–O–C and C–OH bonds in addition to CH and OH bonds. This suggested that carbon-oxygen reaction retarded the formation of stable carbon film for the low friction.

The role of water in the generation of the carbon transfer film may be similar to the mechanisms described for low partial pressure of water in a near vacuum environment and in dry nitrogen [8,9,23]. At a much higher humidity level, the coefficient does not decrease [27,30]. It would be important to find the maximum water concentration at which the coefficient of friction stabilizes to levels below 0.05.

The behavior of ta-C DLC in nitrogen and argon was not as good as that of a-C:H DLC, although it was clear that carbon transfer film was not formed without trace water. From the results of a-C:H DLC in the present study, and from the past studies with hydrogen free DLC where low friction was attained by the introduction of water vapor in the inert gas [13,15,31], it was expected that ta-C DLC would also show friction coefficient as low as 0.1 with trace water. However, ta-C DLC showed friction as high as 0.8 and severe wear, even though the carbon transfer films with CH, OH, C–O–C, and C–OH bonds were formed with 45 ppm and 120 ppm water. On the whole, ta-C DLC shows very high friction and is not suited to be used in inert environments with no trace water or with water at the ppm level.

## 5. Conclusions

Sliding experiments with a-C:H DLC and ta-C DLC and aluminum were conducted in hydrogen, nitrogen, and argon with trace water and no trace oxygen, and the following conclusions were drawn:

(1) The effect of trace water on friction of two types of DLCs in hydrogen was different from that in nitrogen and argon. Trace water of several tens of ppm slightly increases friction in hydrogen, but it decreases the coefficient of friction in nitrogen and argon, particularly with a-C:H DLC.
(2) In hydrogen, both DLCs form transfer films of structured amorphous carbon on the aluminum pin by sliding. The transfer films were formed with and without trace water, and FT-IR spectra indicated that they did not contain oxygen. However, no clear difference in the graphitic structure could be identified by Raman spectroscopy in this study.
(3) In the inert gases of nitrogen and argon with no trace water, no carbon transfer film was formed, which resulted in very high friction. In the sliding between a-C:H DLC and aluminum, trace water of concentrations of 60 ppm and 120 ppm caused a gradual decrease in friction by the formation of carbon transfer films. The transfer films contained CH and OH bonds in nitrogen, and CH, OH, C–O–C, and C–OH bonds in argon.
(4) In nitrogen and argon, ta-C DLC showed friction as high as 0.8 and severe wear with and without trace water, although the carbon transfer films with CH, OH, C–O–C, and C–OH bonds were formed with 45 ppm and 120 ppm water.

## Figures and Tables

**Table 1:** Properties of the DLC coatings.

Types of DLC	a-C:H DLC	ta-C DLC
Hydrogen concentration, atm%	30–40	0–5
Vickers hardness, HV (with 10 mN load)	1200–1500	2000–2300
Surface roughness Ra, μm	0.003	0.02

**Table 2:** Peaks found in FT-IR spectra on pins slid against a-C:H DLC.

Gas	Water	Wavenumbers and Structures									
		3400	3300	2960, 2870	2920, 2840	1610–1640	1540	1410–1420	1150–1275	1000–1100	800–920
		OH	NH	CH_3_	CH_2_	C=C	NH	=CH	C–O–C	C–OH	C–O–C
H_2_	0 ppm	×	×	×	○	×	×	×	×	×	×
	60 ppm	×	×	×	○	×	×	×	×	×	×
	160 ppm	×	×	×	○	×	×	×	×	×	×
N_2_	0 ppm	×	×	×	×	×	×	×	×	×	×
	60 ppm	○	×	○	○	○	×	×	×	×	×
	120 ppm	×	○	○	○	×	○	×	×	×	×
Ar	1 ppm	×	×	×	×	×	×	×	×	×	×
	60 ppm	○	×	○	○	○	×	○	○	○	○
	120 ppm	○	×	○	○	○	×	○	○	○	○

**Table 3:** Peaks found in FT-IR spectra on pins slid against ta-C DLC.

Gas	Water	Wavenumbers and Structures									
		3400	3300	2960, 2870	2920, 2840	1610–1640	1540	1410–1420	1150–1275	1000-1100	800–920
		OH	NH	CH_3_	CH_2_	C=C	NH	=CH	C–O–C	C–OH	C–O–C
H_2_	0 ppm	×	×	×	○	×	×	×	×	×	×
	45 ppm	×	×	○	○	×	×	×	×	×	×
	120 ppm	×	×	○	○	×	×	×	×	×	×
N_2_	0 ppm	×	×	×	×	×	×	×	×	×	×
	45 ppm	○	×	○	○	○	×	○	○	x	○
	100 ppm	○	×	○	○	○	×	○	○	x	○
Ar	0 ppm	×	×	×	×	×	×	×	×	×	×
	45 ppm	○	×	○	○	○	×	○	×	×	○
	120 ppm	○	×	○	○	○	×	○	×	○	○
